# Clinical and microbiological characteristics of bloodstream infections due to AmpC β-lactamase producing Enterobacteriaceae: an active surveillance cohort in a large centralized Canadian region

**DOI:** 10.1186/s12879-014-0647-4

**Published:** 2014-12-14

**Authors:** Vikas P Chaubey, Johann D D Pitout, Bruce Dalton, Daniel B Gregson, Terry Ross, Kevin B Laupland

**Affiliations:** Department of Community Health Sciences, University of Calgary, TRW Building 3rd Floor, 3280 Hospital Drive NW, Calgary, T2N 4Z6 AB Canada; Department of Medicine, Administration Office, Foothills Medical Centre - North Tower, University of Calgary, 9th Floor, 1403 - 29th Street NW, Calgary, T2N 2T9 AB Canada; Department of Pathology and Laboratory Medicine, University of Calgary, Diagnostic & Scientific Centre 9, 3535 Research Road NW, Calgary, T2L 2K8 AB Canada; Department of Microbiology, Immunology and Infectious Diseases, Health Research Innovation Centre, University of Calgary, Room 4AA06, 3330 Hospital Drive NW, Calgary, T2N 4N1 AB Canada; Calgary Laboratory Services, Alberta Health Services, Diagnostic & Scientific Centre 9, 3535 Research Road NW, Calgary, T2L 2K8 AB Canada; Centre for Antimicrobial Resistance, University of Calgary, 3330 Hospital Drive N.W., Calgary, T2N 4N1 AB Canada; Department of Pharmacy Services, Alberta Health Services, Foothills Medical Centre, Rm 1080, 10th floor, 1403 29th St NW, Calgary, T2N 2T8 AB Canada; FRCPC, MDCM, TRW Building, 3rd Floor, 3280 Hospital Drive NW, Calgary, T2N 4Z6 AB Canada

**Keywords:** Beta-lactamases, Bacteremia, Enterobacteriaceae

## Abstract

**Background:**

The objective of this study was to describe the clinical and microbiological characteristics of bloodstream infections (BSIs) due to AmpC producing Enterobacteriaceae (AE) in a large centralized Canadian region over a 9-year period.

**Methods:**

An active surveillance cohort design in Calgary, Canada.

**Results:**

A cohort of 458 episodes of BSIs caused by AE was assembled for analysis. The majority of infections were of nosocomial origin with unknown sources. *Enterobacter* spp. was the most common species while BSIs due to *Serratia* spp. had a significant higher mortality when compared to other AE. Delays in empiric or definitive antibiotic therapy were not associated with a difference in outcome. However, patients that did not receive any empiric antimicrobial therapy had increased mortality (3/5; 60% vs. 57/453; 13%; p = 0.018) as did those that did not receive definitive therapy (6/17; 35% vs. 54/441; 12%; p = 0.015).

**Conclusions:**

Delays in therapy were not associated with adverse outcomes although lack of active therapy was associated with increased mortality. A strategy for BSIs due to AE where β-lactam antibiotics (including oxyimino-cephalosporins) are used initially followed by a switch to non-β-lactam antibiotics once susceptibility results are available is effective.

## Background

Enterobacteriaceae with inducible cephalosporinases such as *Enterobacter cloacae, Citrobacter freundii, Serratia marcescens, Morganella morganii, and Providencia stuartii*, may become resistant to oxyimino-cephalosporins (i.e. cefotaxime, ceftazidime), 7-α-methoxy-cephalosporins (i.e. cephamycins such as cefoxitin) and monobactams by overproducing their chromosomal AmpC β-lactamases [1]. In *Klebsiella pneumoniae*, *Salmonella* spp., and *Proteus mirabilis*, that lack chromosomal β-lactamases, this type of resistance can be mediated by plasmid encoded or imported AmpC cephalosporinases [2]. *Escherichia coli* possesses genes encoding for chromosomal non-inducible AmpC β-lactamases that are regulated by weak promoters and strong attenuators resulting in low amounts of the cephalosporinase [3]. Occasionally, cephamycin-resistant *E. coli* can also carry plasmid-mediated AmpC β-lactamases [2].

There is controversy about the optimal antibiotic therapy for severe infections caused by AmpC producing Enterobacteriaceae (AE), particularly with regards to those that are chromosomally mediated because of the potential for inducibility and selection of derepressed mutants [4]-[7]. Clinical and Laboratory Standards Institute (CLSI) recommends reporting β-lactam susceptibilities of these organisms, with a footnote suggesting that resistance may develop during therapy [8]. Literature has shown the importance of early appropriate antibiotic therapy for severe infections in preventing mortality, [9] but few studies have looked at serious infections caused by AE especially those with chromosomal enzymes. Cephalosporin resistant Enterobacteriaceae have been isolated from patients that have previously received broad-spectrum cephalosporins, [10]-[12] and emergence of cephalosporin resistance has been described during therapy with β-lactams [13],[14]. These studies have focused on a single species of AE, [10]-[12],[14] or have included a limited range of organisms [13]. In addition, these studies have included various types of infections and have not clearly demonstrated that therapy with β-lactams is associated with an increase in mortality.

The objective of this study is to describe the clinical and microbiological characteristics of bloodstream infections (BSIs) due to AE in a large centralized Canadian region over a 9-year period (2000–08). We also investigate the effects of various empiric and definitive antimicrobial treatment regimens on the mortality of patients with BSIs due to AE. Specifically, we look to determine if definitive treatment with β-lactam antibiotics are associated with increased mortality in infections caused by AE.

## Methods

### Study population

The Calgary Zone of Alberta Health Services (previously known as the Calgary Health Region) administers virtually all medical and surgical care to the residents of the cities of Calgary and Airdrie and a large surrounding area (population 1.2 million) in the province of Alberta, Canada. All patients with BSIs caused by AE from January 1, 2000 to December 31^st^, 2008 in the Calgary Zone were included in the study. The Conjoint Health Research Ethics Board at the University of Calgary approved this study and granted a waiver of individual patient consent.

We excluded episodes with incomplete data, polymicrobial episodes and those in which the patients died within 24 hours (which may have precluded their receiving appropriate therapy). To investigate the effects of various empiric and definitive antimicrobial treatment regimens on the mortality of patients, patients were divided into two groups. Group I included patients that received definitive therapy with β-lactams that are hydrolyzed by AmpC β-lactamases (i.e. piperacillin-tazobactam), oxyimino-cephalosporins (i.e. cefotaxime, ceftriaxone, ceftazidime) and monobactams (i.e. aztreonam). Group II included patients that received definitive therapy with non-β-lactams and/or β-lactams with sufficient activity in spite of AmpC β-lactamases (i.e. cefepime and carbapenems).

### Study protocol

An active surveillance cohort design was utilized. Surveillance for AE were identified using the Electronic Surveillance System at Calgary Laboratory Services [15]. International Statistical Classification of Disease and Health Related Problems (ICD) codes were translated into Charlson Comorbidity Scores using standardized algorithms [16],[17]. Antimicrobial therapies were determined using a regional pharmacy database. AE BSIs were defined by the isolation of such bacteria from at least one set of aseptically obtained blood cultures. Repeat isolation from blood from the same patient within 365 days was considered to be the same incident infection.

### Definitions

Empiric therapy was defined as that therapy initiated prior to the availability of blood culture results and sensitivities. Time to first therapy was defined as the time from blood culture draw to receipt of the first dose of antibiotics. Adequate therapy was defined by the receipt of a standard parenteral dose of an antimicrobial to which the organism was susceptible *in vitro* or a standard oral dose of an antimicrobial with sufficient bioavailability, to which the organism was susceptible *in vitro*. If the patient received two drugs from different classes to which the bacterium tested susceptible to one agent but resistant to the other agent, the therapy was considered to be adequate. Adequacy of combination antibiotic therapy required the microorganism to be susceptible to all drugs in the combination. Definitive therapy was defined as the final antibiotic chosen to complete a treatment course after the availability of antimicrobial susceptibilities. If the patient was given adequate antibiotics before or at the time of acquiring the blood culture, the time to first adequate therapy was recorded as zero hours. Mortality was defined as the 30-day mortality of patients as determined by searching provincial databases. Definitions of nosocomial, healthcare associated and community-acquired infections used in this study, have previously been described [18].

### Laboratory methods

Clinical isolates were identified and minimum inhibitory concentrations (MICs) were determined by Vitek 2™ (Vitek AMS; bioMérieux Vitek Systems Inc., Hazelwood, MO). Susceptibility testing and reporting was performed according to the CLSI guidelines for broth dilution [8]. Enterobacteriaceae potentially possessing chromosomally mediated AmpC β-lactamases did not undergo confirmatory testing for the AmpC gene and/or inducibility of these genes.

These included: *Citrobacter* spp*. (C. braakii, C. freundii, C. youngae)*, *Enterobacter* spp*. (E. aerogenes, E. asburiae, E. cancerogenus, E. cloacae, E. sakazakii)*, *Hafnia alvei, Morganella morganii, Providencia* spp*. (P. rettgeri, P. stuartii)* and *Serratia* spp*. (S. liquefaciens, S. marcescens, S. odorfera)* [5].

After isolates were first shown to be non-susceptible to cefoxitin, the presence of AmpC β-lactamases in *E. coli*, *K. pneumoniae* and *Salmonella* isolates was confirmed using previously described boronic acid methodology [19].

The policy of the centralized microbiology laboratory is to suppress the susceptibility results of all β-lactam antibiotics for potential AE. Only fluoroquinolones, aminoglycosides and trimethoprim/sulfamethoxazole are routinely reported for infections caused by potential AE. Cefepime and carbapenem susceptibilities are available upon request.

### Statistical analysis

Analysis was performed using Stata version 10.0 (Stata Corp, College Station, TX). Non-normally distributed data were described using medians with interquartile ranges (IQR). Categorical data were compared using the two-sided Fisher’s exact test. P-values less than 0.05 were considered significant. Logistic regression models were developed to assess the independent effects of various factors on mortality. Treatment adequacy and timing, and all factors found to be significant to the P < 0.1 level in univariate analyses were included in the initial models. Backward stepwise variable elimination was then performed to develop the most parsimonious model. Discrimination was assessed using the area under the receiver operator characteristic (ROC) curve and calibration using the Hosmer-Lemeshow goodness-of-fit test.

## Results

### Study population

Five hundred and ninety incident episodes of AE BSIs were identified between 2000 and 2008. After excluding patients with incomplete data (n = 64), polymicrobial infection (n = 54, 9) or that died within 24 hours of culture draw (n = 14) a cohort of 458 (78%) episodes remained for further analysis.

### Clinical characteristics (see Table 1for further details)

**Table 1:** **Clinical and microbiological characteristics of bloodstream infections due to AmpC-producing Enterobacteriaceae in the Calgary Zone**

	*Enterobacter spp.* ^1^	*Serratia spp.* ^2^	*Citrobacter spp.* ^3^	*Providencia & Morganella spp.* ^4^	*E. coli* ^5^	Other ^6^
No. of episodes	255	73	51	39	30	10
Source of infection (no. episodes)						
Biliary	46	2	6	2	3	4
Bowel	18	5	4	4	2	0
Urinary	43	10	13	15	5	2
Pneumonia	14	13	2	0	1	0
CVS source	0	1	0	0	0	0
SST source	7	4	0	0	0	0
BJ source	1	0	2	4	0	0
CNS source	1	0	0	0	0	0
Unknown	125	38	24	14	19	4
Comorbid conditions						
Myocardial infarction	15 (5.9%)	4 (5.5%)	4 (7.8%)	4 (10%)	2 (6.7%)	0 (0%)
CHF	32 (13%)	18 (25%)	5 (9.8%)	6 (15%)	2 (6.7%)	1 (10%)
PVD	6 (2.4%)	7 (9.6%)	1 (2.0%)	4 (10%)	2 (6.7%)	1 (10%)
CVD	8 (3.1%)	5 (6.8%)	1 (2.0%)	2 (5.1%)	0 (0%)	1 (10%)
Dementia	2 (<1%)	0 (0%)	2 (3.9%)	3 (8.5%)	1 (3.3%)	0 (0%)
CPD	20 (7.8%)	13 (18%)	2 (3.9%)	5 (13%)	8 (27%)	3 (30%)
Rheumatic disease	2 (<1%)	0 (0%)	1 (2.0%)	3 (8.5%)	0 (0%)	0 (0%)
Peptic ulcer disease	9 (3.5%)	4 (5.5%)	1 (2.0%)	2 (5.1%)	0 (0%)	1 (10%)
Hemiplegia/Paraplegia	6 (2.4%)	4 (5.5%)	2 (3.9%)	3 (8.5%)	1 (3.3%)	0 (0%)
Renal disease	31 (12%)	17 (23%)	7 (14%)	9 (23%)	2 (6.7%)	0 (0%)
Mild liver disease	10 (3.9%)	1 (1.4%)	1 (2.0%)	0 (0%)	0 (0%)	0 (0%)
Moderate severe liver disease	12 (4.7%)	4 (5.5%)	1 (2.0%)	2 (5.1%)	2 (6.7%)	1 (10%)
Malignancy	53 (21%)	8 (11%)	15 (29%)	2 (5.1%)	1 (3.3%)	2 (20%)
Metastatic solid cancer	27 (11%)	3 (4.1%)	2 (3.9%)	1 (2.6%)	5 (17%)	1 (10%)
DM w/o complication	25(9.8%)	7 (9.6%)	2 (3.9%)	4 (10%)	3 (10%)	0 (0%)
DM w/ complications	14 (5.5%)	6 (8.2%)	3 (5.9%)	6 (15%)	1 (3.3%)	0 (0%)
Median Charlson (IQR)	3 (1–5)	4 (2–6)	3 (2–4)	4 (3–7)	4 (1–5)	4 (4–6)
Median age (IQR)	58.0 (39 – 72.3)	65.6 (52.1 – 74.2)	59.1 (36.6 – 75.3)	72.9 (62.9 – 79.6)	58.6 (41.1 – 77.3)	71.2 (64.4 – 81.6)
Male sex (%)	62%	60%	75%	74%	33%	50%
Surveillance definition						
Nosocomial	138 (24, 17% )	45 (14, 31%)	22 (1, 4.5%)	13 (2, 15%)	11 (0, 0%)	3 (0, 0%)
HCAI	70 (6, 8.6%)	12 (1, 8.3%)	16 (0, 0%)	17 (3, 18%)	10 (1, 10%)	4 (0, 0%)
Community acquired	47 (3, 6.4%)	16 (3, 19%)	13 (0, 0%)	9 (1, 11%)	9 (0, 0%)	3 (1, 33%)
p-value	0.08	0.248	1.000	1.000	0.633	0.600
Per year of infection						
2000	20 (7.8%)	6 (8.2%)	2 (3.9%)	7 (18%)	0 (0%)	1 (10%)
2001	26 (10%)	13 (18%)	3 (5.9%)	0 (0%)	4 (13%)	3 (30%)
2002	24 (9.4%)	5 (6.9%)	5 (9.8%)	1 (2.6%)	4 (13%)	1 (10%)
2003	33 (13%)	3 (4.1%)	3 (5.9%)	9 (23%)	5 (17%)	2 (20%)
2004	31 (12%)	9 (12%)	8 (16%)	3 (7.7%)	2 (6.7%)	2 (20%
2005	20 (7.8%)	14 (19%)	8 (16%)	8 (21%)	2 (6.7%)	1 (10%)
2006	24 (9.4%)	6 (8.2%)	5 (9.8%)	5 (13%)	7 (23%)	0 (0%)
2007	31 (12%)	7 (9.6%)	10 (20%)	2 (5.1%)	2 (6.7%)	0 (0%)
2008	46 (18%)	10 (14%)	7 (14%)	4 (10%)	4 (13%)	0 (0%)
Susceptibility testing (percentage susceptible)						
Ciprofloxacin	248/255 (98%)	71/73 (97%)	47/50 (94%)	36/38 (95%)	24/30 (80%)	10/10 (100%)
Gentamicin	251/255 (99%)	72/73 (99%)	45/50 (90%)	34/38 (90%)	23/30 (77%)	10/10 (100%)
Piperacillin-tazobactam	203/255 (80%)	69/73 (95%)	46/50 (92%)	38/38 (100%)	29/30 (97%)	7/10 (70%)
TMP/SMX	242/255 (95%)	73/73 (100%)	39/50 (78%)	31/38 (82%)	19/30 (63%)	10/10 (100%)
Tobramycin	251/255 (99%)	64/73 (88%)	45/50 (90%)	33/38 (87%)	26/30 (87%)	10/10 (100%)
Imipenem	255/255 (100%)	73/73 (100%)	50/50 (100%)	38/38 (100%)	30/30 (100%)	10/10 (100%)

^1^Includes 34 *E. aerogenes,* 1 *E. asburiae,* 2 *E. cancerogenus*, 210 *E. cloacae,* 6 *E. sakazakii* and 2 *E. spp* bacteremia episodes.

^2^Includes 3 *S. liquefaciens,* 67 *S. marcescens,* 1 *S. odorfera* and 2 *S. spp.* bacteremia episodes.

^3^Includes 17 *C. braakii,* 30 *C. freundii,* 2 *C. spp.* and 2 *C. youngae* bacteremia episodes.

^4^Includes 27 *M. Morganii*, 3 *P. rettgeri* and 9 *P. stuartii* bacteremia episodes.

^5^Includes 30 E. coli bacteremia episodes.

^6^Includes 7 *H. alveii*, 2 *Salmonella spp.* and 1 *K. pneumoniae* bacteremia episodes.

CVS = Cardiovascular, SST = Skin and Soft Tissue, BJ = Bone and Joint, CNS = Central Nervous System, CHF = Congestive Heart Failure, PVD = Peripheral Vascular Disease, CVD = Cerebrovascular Disease, CPD = Chronic Pulmonary Disease, TMP/SMX = Trimethoprim/Sulfamethoxazole.

The median patient age was 62.5 years (IQR, 43.6-75.6 years), 283 (62%) were male, and the median age-adjusted Charlson score was 4 (IQR, 4–11). The majority of infections were nosocomially-acquired (n = 232, 51%) and patients most often presented with primary BSIs (n = 224, 50%). The most common comorbid condition was malignancy (n = 81, 18%). BSIs due to AE remained stable over the time period; however we did notice a slight increase in 2008.

### Microbiological characteristics (see Table 1for further details)

*Enterobacter* (n = 225, 49%), *Serratia* (n = 73, 16%) and *Citrobacter* (n = 51, 11%) species were the most common bacteria responsible for BSIs due to AE. Ciprofloxacin, gentamicin, tobramycin, cefepime and imipenem showed excellent activity against various AE (excluding *E. coli)* with susceptibility ranging from 89%-100%. *E. coli* with AmpC β-lactamases showed the highest levels of resistance to ciprofloxacin (20%), gentamicin (23%), and tobramycin (13%). Excluding primary BSIs, there was a strong association of *Enterobacter* spp. with the presence of underlying BTIs (46/255, 18%) while the isolation of *Citrobacter* and *Providencia/Morganella* spp were associated with underlying UTIs.

### Outcomes (see Table 2for further details)

**Table 2:** **Factors investigated for association with 30-day case fatality (univariate analysis)**

Factor	Case fatality with factor	Case fatality without factor	Relative-risk (95% ci)	P-value
Male	42/283 (15%)	18/175(10%)	1.44 (0.86-2.42)	0.20
*Age-adjusted Charlson > median (4)	48/230 (21%)	12/228 (5.3%)	3.97 (2.16-7.26)	<0.001
Source of bacteremia				0.001
Unknown	26/224 (12%)			
Urinary	6/88 (6.8%)			
Biliary	4/63 (6.3%)			
Bowel	7/33 (21.1%)			
Pneumonia	12/30 (40%)			
Skin and soft tissue infection	3/11 (28%)			
Bone and joint	2/7 (29%)			
CNS	0/1 (0%)			
CVS	0/1 (0%)			
Location of acquisition				0.015
Nosocomial	41/232 (17.7%)			
Healthcare associated	11/129 (8.5%)			
Community-acquired	8/97 (8.2%)			
Microbiology**				0.012
*Enterobacter spp.*	33/255 (13%)			
*E. aerogenes*	5/34 (15%)			
*E. asburiae*	0/1 (0%)			
*E. cancerogenus*	0/2 (0%)			
*E. cloacae*	26/210 (13%)			
*E. sakazakii*	2/6 (33%)			
*E. spp.*	0/2 (0%)			
*Serratia spp.*	18/73 (25%)			
*S. liquefaciens*	2/3 (66%)			
*S. marcescens*	15/67 (22%)			
*S. odorfera*	1/1 (100%)			
*S. spp.*	0/2 (0%)			
Citrobacter spp.**	1/51 (2.0%)			
*C. braakii*	0/17 (0%)			
*C. freundii*	1/30 (3.3%)			
*C. spp.*	0/2 (0%)			
*C. youngae*	0/2 (0%)			
*Escherichia coli*	1/30 (3.3%)			
*Morganella morganii*	4/27 (15%)			
*Providencia spp.*	2/12 (17%)			
*P. rettgeri*	1/3 (33%)			
*P. stuartii*	1/9 (11%)			
*Hafnei alvei*	1/7 (14%)			
*Salmonella spp.*	0/2 (0%)			
*Klebsiella pneumoniae*	0/1 (0%)			
Plasmid mediated	1/33 (3.0%)	59/425 (14%)	0.22(0.03-1.53)	0.10
Description of empiric therapy				0.12
Empiric therapy in hours at:				
t = 0	23/172 (13%)			
t > 0 and t = 8	11/105 (10%)			
t >8 and t = 24	17/114 (15%)			
t > 24 and t = 48	3/36 (8.3%)			
Empiric at t > 48 hrs.	3/26 (12%)			
Never received empiric therapy.	3/5 (60%)			
Description of adequate therapy				0.05
Adequate therapy in hours at:				
t = 0 hrs.	11/98 (11%)			
t > 0 and t = 8	11/96 (11%)			
t >8 and t = 24	23/133 (17%)			
t > 24 and t = 48	5/62 (8.1%)			
t > 48 hrs.	4/52 (7.7%)			
Never received adequate or definitive therapy.	6/17 (35%)			

CNS: Central Nervous System. CVS: Cardiovascular System. 95% CI: 95% Confidence Interval.

*Charlson data was missing for 11 patients; their Charlson score was assumed to be zero and then adjusted for age.

**Some isolates were not speciated beyond the genus level.

Overall 60 (13%) patients died within thirty days. *S. marcescens* had the highest mortality (15/52; 29%) and *Citrobacter* spp. had the lowest mortality (1/51; 2.0%).

### Description of therapy (see Tables 2 and 3for further details)

**Table 3:** *Empiric, first adequate and definitive antimicrobial choice and associated 30-day case-fatality

Empiric antibiotic choice	Case fatality rate	First adequate antibiotic choice	Case fatality rate	Definitive antimicrobial therapy	Case fatality rate
*Antibiotics with activity against AmpC- producing Enterobacteriaceae*					
Aminoglycoside	7/52 (13%)	Aminoglycoside	10/80 (13%)	Aminoglycoside	15/132 11%)
Beta-lactam/Beta-lactamase inhibitor combination	15/131 (11%)	Beta-lactam/Beta-lactamase inhibitor combination	16/137 (12%)	Beta-lactam/Beta-lactamase inhibitor combination	10/22 (45%)
Carbapenem	0/16 (0%)	Carbapenem	3/31 (9.7%)	Carbapenem	5/45 (11%)
Cefepime	1/3 (33%)	Cefepime	1/5 (20%)	Cefepime	1/4 (25%)
Cefoxitin	0/1 (0%)	Cefixime	0/10 (0%)	Cefotaxime	0/2 (0%)
Ceftazidime	1/15 (6.7%)	Ceftazidime	0/10 (0%)	Ceftazidime	0/1 (0%)
Ceftriaxone	11/85 (13%)	Ceftriaxone	12/97 (12%)	Ceftriaxone	1/7 (14%)
Cefuroxime	1/5 (20%)	Colistin	0/1 (0%)	Colistin	0/1 (0%)
Colistin	0/1 (0%)	Fluoroquinolone	16/106 (15%)	Fluoroquinolone	30/231 (13%)
Fluoroquinolone	10/76 (13%)	TMP/SMX	0/9 (0%)	TMP/SMX	0/14 (0%)
TMP/SMX	2/14 (14%)				
*Antibiotics unlikely to have activity against AmpC- producing Enterobacteriaceae*					
Ampicillin	3/30 (10%)				
Azithromycin	1/5 (20%)				
Cefazolin	5/39 (13%)				
Cephalexin	0/2 (0%)				
Clindamycin	0/4 (0%)				
Cloxacillin	2/7 (29%)				
Linezolid	0/1 (0%)				
Metronidazole	12/68 (18%)				
Penicillin	0/3 (0%)				
Rifampin	0/3 (0%)				
Vancomycin	6/37 (16%)				

TMP/SMX = Trimethoprim/Sulfamethoxazole.

*For each category the total number of episodes adds up to more than the actual number of episodes because some patients received multiple antibiotics corresponding to each time period.

Empiric therapy was provided in 453 (99%) cases. The most common agents used as empiric therapy included piperacillin-tazobactam (131, 29%) and oxyimino-cephalosporins (106, 23%). Empiric therapy was started immediately in 172 (38%) patients, within 8 hours in 277 (60%) patients, within 24 hours in 391 (85%) patients and within 48 hours in 427 (93%) patients. The timing of empiric antibiotic therapy was not associated with a difference in outcome. However, patients that did not receive empiric antimicrobial therapy had a worse outcome (3/5; 60% vs. 57/453; 13%; p = 0.018). The number, combination (at least two antibiotics from different classes), or class of antibiotic was also not associated with any statistically significant improvement in outcome (data not shown). Empiric therapy with piperacillin-tazobactam or an oxyimino-cephalosporin was not associated with a difference in mortality outcome (28/237; 12% vs. 32/221; 14%; p = 0.41). Of 131 that received empiric piperacillin-tazobactam, the case-fatality rate was 2/19 (11%) for those patients with isolates that tested resistant at baseline as compared to 13/112 (12%) for those isolates that tested sensitive, p = 0.89. Notably, of 106 patients who received oxyimino-cephalosporins as empiric therapy, the case-fatality rate was 5/18 (28%) for those patients with isolates that tested resistant at baseline as compared to 8/88 (9%) for those that were sensitive, p = 0.04.

Adequate therapy was provided in 441 (96%) cases. The most common agents used as adequate therapy included piperacillin-tazobactam (133, 29%) and oxyimino-cephalosporins (106, 23%). Adequate therapy was received immediately in 98 (21%) patients, within 8 hours in 194 (42%) patients, within 24 hours in 327 (71%) patients and within 48 hours in 389 (85%) patients. Early receipt of adequate antibiotic therapy was not associated with improved outcomes. Adequate number, combination (at least two antibiotics from different classes to which the microorganism had in vitro susceptibility) or class of antibiotic was not associated with improved outcome (data not shown). First adequate therapy with piperacillin-tazobactam or an oxyimino-cephalosporin was not associated with different outcomes (28/241; 12% vs. 32/217; 15%; p = 0.33). Patients that did not receive any form of definitive therapy did have a worse outcome (6/17; 35% vs. 54/441; 12%; p = 0.015). The analysis that assessed the outcomes of patients in groups I and II did not reveal a mortality difference (Group I: 4/22; 18% vs. Group II: 50/419; 12%, p = 0.33) or a differences in relapse of infection with the same bacteria (Group I: 3/22; 14% vs. Group II: 27/419, 6.4%, p = 0.182).

### Logistic regression model (see Table 4for further details)

**Table 4:** **Logistic regression model**

Variable	Odds ratio	95% CI	P-value
Age adjusted Charlson score (per point)	1.34	1.20-1.49	<0.001
Source of infection			
Biliary (reference)			
Bowel	3.87	1.27-11.80	0.018
Pneumonia	12.98	4.49-37.46	<0.001
SSTI	12.64	2.35-68.07	0.003
Unknown	2.24	1.04-4.822	0.039
Treatment group			
Adequate therapy at T = 8-24 hours.	2.01	1.00-4.03	0.050
Adequate therapy at T > 24 hours to T = 48 hours.	0.44	0.18-1.07	0.70
Never received definitive therapy.	4.78	1.39-16.44	0.013

A logistic regression model was developed to assess the effect of timing and adequacy of therapy (n = 458) and had good calibration (Hosmer-Lemeshow goodness of fit p = 0.49 and discrimination (area under ROC = 0.808; ROC curve not shown). Early adequate therapy between 8–24 hours after blood culture draw had worse outcomes (Odds Ratio (OR) 2.01, p = 0.050) while delayed therapy at T > 24 hours to T = 48 hours had increased survival that did not reach statistical significance (OR 0.44, p = 0.70). Never having received definitive therapy demonstrated an increased risk of death (OR 4.78, p = 0.013).

## Discussion

Our study described the clinical and microbiological characteristics of BSIs due to AE over a 9-year period. The majority of infections were of nosocomial origin with unknown sources. Patients often had underlying malignancy and the highest rates of mortality were associated with underlying pneumonia. *Enterobacter* spp. was by far the most common bacteria responsible for BSIs due to AE and was associated with underlying BTIs. Interestingly, we found that BSIs due to *Serratia* spp. had a significant higher mortality when compared to other AE.

As demonstrated in our study, previous studies of AE BSIs have consistently shown *Enterobacter* spp. as the most common microbiologic etiology, [20]-[23] and malignancy as the most common underlying condition [10],[21]-[29]. The most common sources of AE BSIs from the literature include unknown source, biliary source and urinary source (variably ranked), in studies including either a single species or multiple species of AE [10],[23]-[27]. Our study is unique in that the mortality of *Serratia* spp. BSIs is particularly high relative to what others have found [21].

Early and adequate antimicrobial therapy is a cornerstone of treatment of severe infections including BSIs. Several studies have found a clear relationship between time to first adequate therapy and mortality [9],[30]-[32]. Most of these studies utilize *in vitro* susceptibility to establish adequacy of therapy and this may not be appropriate in the specific case of AE organisms. Our study revealed that delays in active antibiotic therapy were not associated with a poor outcome. However, patients that did not receive any empiric or definitive antimicrobial therapy had increased mortality. While the importance of appropriate early antimicrobial therapy is generally accepted, there are several studies that do not support this premise [33]-[37]. Our patient population was not limited to the critically ill and this may explain why we were not able to demonstrate an association between early adequate antimicrobial therapy and increased survival.

Previous studies have investigated the issue of resistance development during treatment with oxyimino-cephalosporins of infections due to AE. A previous study had shown a low risk of emergence of resistance during therapy and a low risk of associated mortality [13]. However that study included only a select number of AE and included a broad range of infections types. Another study showed that after treatment, 477 patients with susceptible *Enterobacter* spp*.* infections, 49 (10.3%) subsequently developed cephalosporin resistant isolates [11]. This study did not specifically address mortality. The policy of the centralized laboratory to suppress susceptibilities for all β-lactam antibiotics for infections caused by AE resulted in clinicians changing the definitive antimicrobial therapy to non-β-lactam antibiotics once susceptibility results were available. After excluding all patients that died early and that did not receive definitive therapy only 22 of 441 patients completed definitive therapy with oxyimino-cephalosporins. The low numbers of patients treated with oxyimino-cephalosporins thus precluded us from determining the efficacy of this regimen. Our study did however reveal that patients empirically treated with oxyimino-cephalosporins, when the isolate was resistant at baseline, had a statistically worse outcome. This supports the notion that initial empiric therapy with oxyimino-cephalosporins may be effective for AE that test *in vitro* sensitive to these agents. We recommend that patients who previously received oxyimino-cephalosporins should be treated empirically with an alternate class of agent because of the risk of being colonized with oxyimino-cephalosporin resistant AE.

In the multivariate model it was noted that adequate therapy at between 8–24 hours after blood culture draw had worse outcomes while delayed adequate therapy at T > 24 hours to T = 48 hours had increased survival; findings that did not reach statistical significance. Our group has previously reported similar results [34]. This interesting finding was most likely the result of sicker patients receiving broader and more prompt empiric antimicrobials as a result of their clinical condition. Again, our study is limited in that we did not collect data regarding severity of illness. Furthermore, since these findings did not reach statistical significance they may have occurred by chance alone. It should be mentioned that the numbers of patients that did not receive empiric or definitive therapy were small, 5 and 17 respectfully. As such, any specific conclusions could not be generated.

There are several limitations of our study mostly arising from our pharmacy database. This database did not capture all outpatient data. It is conceivable that the 22 (or a proportion thereof) patients that are classified as having completed definitive β-lactam therapy may have received outpatient definitive non-β-lactam therapy after initial improvement. In addition, the pharmacy database may have not captured all ward stock antibiotics dispensed and the pharmacy database captured the time the antibiotic was dispensed which may not have correlated with the time the antibiotic was administered. Finally, the cohort assembled for this study included potential AmpC producers, and it is possible that some of the isolates may not have had the AmpC β-lactamase gene. In the clinical context it is impractical due to time and financial constraints to test each isolate for the presence of the AmpC gene and/or inducibility of these genes. As such our study attempted to answer this question from a feasibility perspective.

## Conclusion

In conclusion, *Enterobacter* spp*.* is the most common AE that causes BSIs in the Calgary region. A strategy where β-lactam therapy (including oxyimino-cephalosporins) is used as initial therapy for severe infections caused by AE followed by a switch to non-β-lactam therapy once susceptibility results are available is effective. The efficacy of using oxyimino-cephalosporins as definitive therapy for severe AE infections remains unknown.

## Abbreviations

BSI: Bloodstream infection
AE: AmpC producing Enterobacteriaceae
CLSI: Clinical and Laboratory Standards Institute
ICD: International Statistical Classification of Disease and Health Related Problems
MIC: Minimum Inhibitory Concentrations
IQR: Interquartile ranges
ROC: Receiver operator characteristic
UTI: Urinary Tract Infections
BTI: Biliary Tract Infections
OR: Odds Ratio
